# RETRACTED: Vlasa et al. Association of *Entamoeba gingivalis* with Periodontal Disease—Systematic Review and Meta-Analysis. *Medicina* 2024, *60*, 736

**DOI:** 10.3390/medicina62030447

**Published:** 2026-02-27

**Authors:** Alexandru Vlasa, Anamaria Bud, Luminita Lazar, Ana Petra Lazar, Alexander Herbert, Eugen Bud

**Affiliations:** 1 Faculty of Dental Medicine, George Emil Palade University of Medicine, Pharmacy, Science, and Technology, 540139 Târgu-Mureș, Romania; alexandru.vlasa@umfst.ro (A.V.); luminita.lazar@umfst.ro (L.L.); ana.lazar@umfst.ro (A.P.L.); eugen.bud@umfst.ro (E.B.); 2 Independent Researcher, 71034 Boblingen, Germany; a.herbert1990@web.de

The journal retracts the article titled, “Association of *Entamoeba gingivalis* with Periodontal Disease—Systematic Review and Meta-Analysis” [1], cited above.

Following publication, concerns were brought to the attention of the Editorial Office regarding the accuracy of the cited sources and errors in the methodology used to conduct this systematic review [1].

In accordance with the journal’s standard procedures, the Editorial Office and Editorial Board conducted an investigation that identified major scientific flaws, including noncompliance with systematic review guidelines, heterogeneity issues, inappropriate bias assessment reporting, inclusion of unreferenced studies, misclassification of unrelated studies, and omission of eligible studies. As a result, the Editorial Board has decided to retract this article as per MDPI’s retraction policy.

This retraction was approved by the Editor-in-Chief of the journal *Medicina*.

The authors have been informed of this retraction and have indicated their disagreement with this decision.
